# Cognitive impairment following breast cancer treatments: an umbrella review

**DOI:** 10.1093/oncolo/oyae090

**Published:** 2024-05-09

**Authors:** Giulia Oliva, Andreina Giustiniani, Laura Danesin, Francesca Burgio, Giorgio Arcara, Pierfranco Conte

**Affiliations:** Department of Surgery, Oncology and Gastroenterology (DiSCOG), University of Padua, 35124 Padova, Italy; IRCCS San Camillo Hospital, 30126 Venice, Italy; IRCCS San Camillo Hospital, 30126 Venice, Italy; IRCCS San Camillo Hospital, 30126 Venice, Italy; IRCCS San Camillo Hospital, 30126 Venice, Italy; IRCCS San Camillo Hospital, 30126 Venice, Italy; IRCCS San Camillo Hospital, 30126 Venice, Italy

**Keywords:** breast cancer, cancer treatments, cognitive impairment, objective cognitive deficits, subjective cognitive concerns, quality of life

## Abstract

**Objectives:**

Cancer-related cognitive impairment (CRCI) refers to a cognitive decline associated with cancer or its treatments. While research into CRCI is expanding, evidence remains scattered due to differences in study designs, methodologies, and definitions. The present umbrella review aims to provide a comprehensive overview of the current evidence regarding the impact of different breast cancer therapies on cognitive functioning, with a particular focus on the interplay among objective cognitive deficits (ie, measured with standardized tests), subjective cognitive concerns, (ie, self-reported), and other mediating psycho-physical factors.

**Methods:**

The search was made in Pubmed, Embase, and Scopus for articles published until July 2023, following the guidelines of the Preferred Reporting Items for Systematic Reviews and Meta-analysis protocol.

**Results:**

Chemotherapy and endocrine therapy appear consistently associated with CRCI in patients with breast cancer, primarily affecting memory, attention/concentration, executive functioning, and processing speed. Subjective cognitive concerns were often found weakly or not associated with neuropsychological test results, while overall CRCI seemed consistently associated with psychological distress, fatigue, sleep quality, and inflammatory and biological factors.

**Conclusion:**

Current evidence suggests that CRCI is common after chemotherapy and endocrine therapy for breast cancer. However, heterogeneity in study designs and the scarcity of studies on more recent treatments such as targeted therapies and immunotherapies, highlight the need for more systematic and harmonized studies, possibly taking into account the complex and multifactorial etiology of CRCI. This may provide valuable insights into CRCI’s underlying mechanisms and potential new ways to treat it.

## Teaser

This review provides a comprehensive overview of the current evidence regarding the impact of different breast cancer therapies on cognitive functioning, with a particular focus on the interplay among objective cognitive deficits, subjective cognitive concerns, and other mediating psycho-physical factors.

**Implications for practice**

Advances in breast cancer treatments have significantly extended patient survival. However, with a growing number of patients experiencing cancer-related cognitive impairment (CRCI), clinicians must now account for the cognitive side effects of chemotherapy, hormonal therapy, and newer therapies. Integrating comprehensive cognitive assessments into routine cancer care can aid in early detection. Furthermore, recognizing CRCI’s impact on daily functioning and its complex association with psychological well-being, fatigue, and overall quality of life, can help clinicians develop personalized interventions and preventive approaches. This would facilitate bridging the gap between cancer care and cognitive health in a holistic patient-centered approach.

## Introduction

Breast cancer (BC) is the most frequently diagnosed cancer and poses a significant public health burden.^1^ Recently, the life expectancy of patients with BC has improved significantly, primarily due to the availability of more effective treatments such as endocrine therapies plus CDK4/6 inhibitors for hormone receptor-positive tumors, antiHER2 drugs for HER2-positive tumors, immunotherapy for triple-negative disease, and PARP inhibitors for BRCA mutated tumors. Moreover, the increased awareness of the importance of mammography screenings led to earlier diagnoses and treatment.^2,3^

The changing landscape of BC treatments and the increasing numbers of long-term survivors have drawn more attention to the adverse effects of cancer therapies, encompassing cardiotoxicity,^4^ the development of second malignancies,^5^ and, after specific endocrine and chemotherapeutic treatments, the occurrence of menopausal symptoms.^6,7^ Persistent pain following BC surgery and radiotherapy is not uncommon either.^8^

In addition, a growing number of studies have found cancer-related cognitive impairment (CRCI) in patients with BC and survivors, with a prevalence ranging from 13% to 70%.^9^ CRCI refers to a cognitive decline associated with cancer itself or its treatments.^10,11^ These deficits pertain mostly to learning and memory, processing speed, executive function, and word retrieval.^9,12^ Furthermore, patients often report subjective cognitive concerns such as forgetfulness, distractibility, and word-finding difficulties.^13^ CRCI can occur during and immediately after the treatment, or with a delayed onset.^14^ Similarly, in some patients, cognitive symptoms are reduced after treatment’s cessation whereas, in other patients, deficits outlast the end of the treatment with a long-term persistence.^15,16^ However, it should be noted that differences in results among studies also depend on the use of different modalities of cognitive assessment. Indeed, a distinction exists between objective cognitive deficits and subjective cognitive concerns. Objective cognitive functioning refers to cognitive data gathered by standardized tests in which patients are asked to perform tasks to the best of their capabilities.^17^ Subjective cognitive functioning refers to patients’ self-report on cognitive abilities or other psychological aspects (eg, stress and anxiety) that may affect cognition.

Objective and subjective cognitive assessments may return highly different results concerning patients’ with BC cognitive status. Interestingly, prospective studies have revealed weak correlations between objective neuropsychological tests’ results and subjective cognitive concerns. In contrast, stronger correlations have been found between subjective concerns and symptoms such as fatigue, sleep disorders, and psychological distress.^18,19^

Moreover, cognitive deficits are negatively influenced by anxiety and mood disorders,^20,21^ body image concerns,^22^ and fear of recurrence^23^ which typically occur in patients with BC, all of which negatively influence cognition.^24^ These factors can collectively contribute to a multifaceted phenomenon in which they interact and mutually influence each other, contributing to the development and persistence of cognitive problems.

In this complex scenario, while it has been recognized that systemic cancer therapies can significantly impact cognition, several gaps persist, necessitating further research to elucidate CRCI, its prevalence among patients with BC, and the underlying bio-psycho-social mechanisms.

### Open issues in cancer-related cognitive impairment research

Albeit in 2011 the International Cancer and Cognition Task Force formulated research guidelines and recommendations to harmonize CRCI research, to date evidence remains limited, with substantial heterogeneity in study protocols and methodologies.^9^

Additionally, most research has primarily focused on the cognitive effects of chemotherapy and hormonal therapies,^25^ while studies on the neurotoxicity of immunotherapies and targeted therapies remain scarce.

Despite the International Cancer and Cognition Task Force’s recommendations for a standardized set of validated neuropsychological tests, there is still no consensus on the cognitive tools that can reliably detect cognitive impairment in this population. The use of different types of neuropsychological tests across studies, coupled with varying definitions of cognitive impairment, has resulted in inconsistent findings.^26,27^

Finally, the limited number of longitudinal studies and the wide array of methods hamper accurate estimations of the incidence and trajectory of post-treatment cognitive decline, and the identification of reliable biomarkers and risk factors.

Given the aforementioned concerns, this umbrella review aims to provide a comprehensive summary of the up-to-date evidence regarding the impact of various cancer therapies used in BC treatment on cognitive functioning. In particular, the review has 3 main goals:

- Investigating objective cognitive functioning, as measured with standardized tests and scales.
- Investigating subjective cognitive functioning, as measured through self-report on cognitive abilities or self-report questionnaires, and exploring the connection between objective and subjective cognitive functioning.
- Investigating the psycho-physical factors (eg, fatigue, stress, sleep quality, anxiety, and depression) that may play a role in this complex relationship.

## Methods

This study was conducted by following the Preferred Reporting Items for Systematic Reviews and Meta-Analyses (PRISMA^28^;). The Scopus, PubMed, and Embase databases were systematically searched from January 2010 to July 2023. Controlled vocabulary, ie, MeSH terms and Emtree, was adopted to include the search terms, considering titles and abstracts. The following search syntax was used: ((“breast cancer” OR “breast neoplasm” OR “mammary cancer”) AND (“chemotherapy” OR “radiotherapy” OR “endocrine therapy” OR “hormonal therapy” OR “immunotherapy”) AND (cognit* OR neuropsycholog*)) AND” systematic review.”

The full search strategy and syntax for each database are reported in Supplementary material Table S1.

To be eligible, studies had to meet the following criteria:

- being systematic reviews;
- assessing patients treated for early BC or BC survivors after the completion of treatment;
- reporting a neuropsychological test as primary or secondary outcomes;
- being written in English;
- being published in an English language journal after 2010.

Candidate studies were excluded when they were published in non-scientific journals, were not conducted on humans, and did not use neuropsychological tests as primary or secondary outcomes. Other reviews were inspected to extract possible eligible papers.

**Figure 1.**
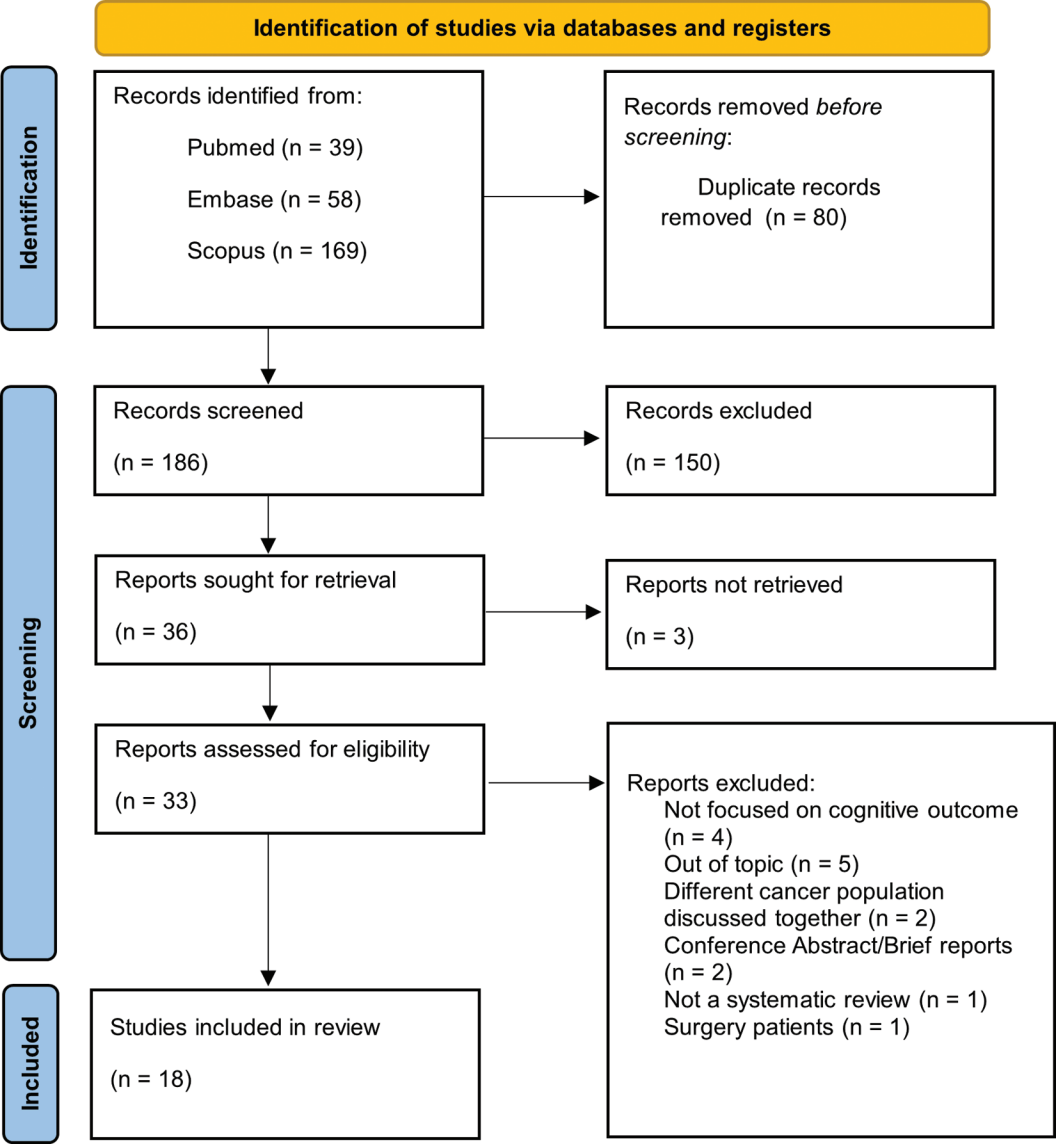
PRISMA flowchart. Preferred Reporting Items for Systematic Reviews and Meta-Analyses (PRISMA) chart illustrating the process for final selection of the included studies.

Three authors (GO, AG, LD) independently screened the titles and abstracts of articles collected from the database search. Only articles meeting the inclusion criteria were selected. Any disagreement in study selection was discussed and resolved among all the authors. Two authors (GO, AG) read the remaining articles and extracted relevant information following a modified version of the PICO guidelines: participants, methodology, comparisons, and outcomes. Additional data on the sample’s demographic were extracted. See Figure 1 for the PRISMA flowchart.

### Quality assessment

Methodological quality and risk of bias were evaluated using the AMSTAR 2,^29^ a critical appraisal tool for systematic reviews that include randomized and non-randomized studies of healthcare interventions (Table S2, Supplementary materials).

## Results

### Description of studies

Eighteen systematic reviews were included in the present study. These focused on cancer stages ranging from I to III, while consistently excluding cases of metastatic cancer. Overall, ages ranged from 18 to >70, involving both pre-menopausal and post-menopausal women. Thirteen reviews focused on chemotherapy, 4 reviews on hormonal therapy, and one considered both.

**Figure 2.**
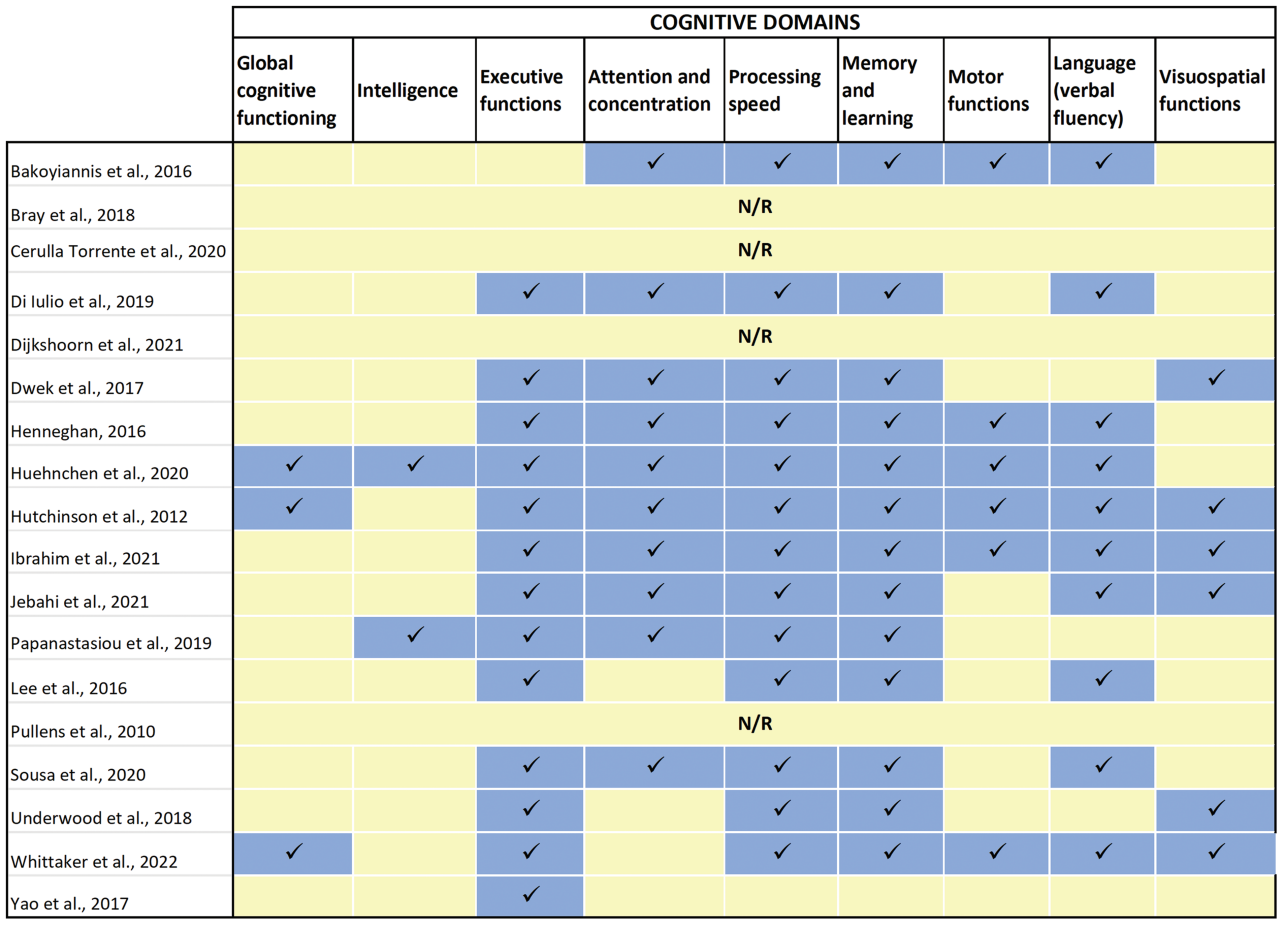
Cognitive domains investigated in each included systematic review. *In blue:* that specific domain has been investigated; *in yellow:* the specific domain was not investigated; *N/R:* not reported. Four systematic reviews did not report details about which cognitive domains were examined in the included primary studies, generally referring to neuropsychological assessment outcome.

CRCI was assessed using objective neuropsychological testing (*n* = 10) or a combination of patients-reported cognitive functioning and objective tests (*N* = 8). The cognitive domains most frequently investigated included memory and learning, executive functions, processing speed, attention/concentration, working memory, motor, and psychomotor speed (Figure 2). Additionally, 7 reviews also explored other related outcomes, such as fatigue (*n* = 3), depression (*n* = 3), anxiety (*n* = 2), stress (*n* = 2), psychological distress (*n* = 2), and quality of life (*n* = 2). The neuropsychological tests and self-report questionnaires used to assess cognitive functioning widely varied across the studies (Table S3, Supplementary material).

Results are organized by the specific outcome of interest. Reviews that investigated multiple clinical outcomes are described separately. See Table 1 for further information on the included systematic reviews.

**Table 1.** Information on included systematic reviews

Authors, year	Included studies (number, study design)	Clinical population	Stage	Molecular type	Sample characteristics	Menopausal state	Cancer therapy	Therapy setting	Pharmacological details	Outcome of interest	Cognitive domains	Other measures of interest	Authos’ summary of results
Bakoyiannis et al, 2016	12—prospective and retrospective observational cohort studies; RCT	BC; HC; Women with BC who did not receive endocrine therapy	Stage I to III	HR+	2756 patients with BC, 375 controls; N/A; 100% females	Pre-menopause; post-menopause	ET	Adjuvant	Tamoxifen (*N* = 7); raloxifene (*N* = 2); anastrazole (*N* = 2); aromatase inhibitors (*N* = 1); exemestane (i = 3); N/A (*N* = 4)	Objective cognitive functioning	Verbal memory, verbal Fluency, attention/working memory, motor speed; psychomotor speed	None	Treatment with ET seems to be accompanied by altered cognitive abilities, including verbal memory, verbal fluency, motor speed, attention, and working memory. Tamoxifen seems to be related to decreased cognitive performances compared with aromatase inhibitors.
Bray et al, 2018	101	Patients with cancer with self-reported CI who received chemotherapy	N/A	N/A	22347 patients (79% BC, 21% other primary tumors); N/A; N/A	Pre-menopause; post-menopause	CT	N/A	N/A	Subjective cognitive functioning	N/A	None	Most studies found a lack of association between self-reported cognitive symptoms and neuropsychological results. A minority of studies reported a significant association between the 2.
Torrente et al, 2020	47—Longitudinal	BC (*N* = 39); colorectal cancer (*N* = 4); testicular cancer (*N* = 3); lung cancer (*N* = 1)	Stage I to III	N/A	5976 total subjects; N/A; N/A	N/A	CT	Adjuvant; neoadjuvant	N/A	Objective cognitive functioning	N/A	None	CRCI was most frequent in patients with BC, with impairment in attention, memory, executive functions, and processing speed. Language, visuospatial and visuoconstructional abilities were preserved, consistently with subjective cognitive complaints. CRCI was more frequent after CT cycles and seemed to improve 1-2 years after treatment.
Iulio et al, 2019	29 (21 on breast cancer)	Different types of non brain cancer	Stage I-III	N/A	1802	N/A	CT, RT, ET	N/A	N/A	Objective cognitive functioning	Attention/concentration; executive function; verbal/visual memory; psychomotor speed; language; processing speed; motor function; planning; verbal fluency; working memory	None	Patients with BC appeared to be the most affected in neuropsychological function after CT. Overall, the most impaired functions were verbal ability, memory, executive function, and motor speed.
Dijkshoorn et al, 2021	17	Patients wit BC	N/A	N/A	N/A	N/A	CT vs ET	N/A	N/A	Objective cognitive functioning	N/A	None	Compared to their pretreatment cognitive functioning, 24% of patients decline after treatment and 24% at 1‐year. Some studies also found that 15% and 31% of patients improve, respectively after treatment.
Dweck et al, 2017	17 (12 on breast cancer)	Solid tumors	N/A	N/A	N/A	N/A	CT	Adjuvant	N/A	Objective cognitive functioning	Processing speed, verbal/ visual memory, working memory, attention, psychomotor functions, executive function, spatial function	Quality of life	There was evidence of cognitive impairment in patients with BC treated with CT. Verbal memory was the most affected cognitive domain, followed by processing speed and executive functioning. However, CRCI did not consistently appear to affect patients’ QoL
Henneghan, 2016	27	Patients with BC	0 to III	N/A	Average sample size: 111.09 (SD 91.05, range 19-317)	N/A	CT	N/A	N/A	Objective and perceived cognitive impairment	Verbal/visual memory; processing speed; response speed; attention; perceived cognitive function; working memory; executive function; motor coordination; psychomotor speed; language	Fatigue	There was evidence of significant relationships between cognitive impairment in various domains and modifiable biological, behavioral, and psychosocial factors, such as inflammatory cytokines, sleep quality, physical activity, stress, distress, and affect.
Huehnchen et al, 2020	30	Patients with BC and others	N/A	N/A	1157	N/A	CT	N/A	Cyclophosphamide, epirubicin or doxorubicin, docetaxel or paclitaxel, doxorubicin, and 5-fluorouracil (FEC or FEC-T regimen)	Objective cognitive functioning	Verbal and visuospatial memory; working memory; reaction time; intelligence; executive function; global cognitive function; processing speed; short-term memory; attention; speech; motor function; visual scanning; semantic fluidity	None	Certain CT regimens were frequently associated with cognitive impairment in breast cancer patients. Particularly, combinations of 5-fluorouracil, epirubicin, cyclophosphamide, doxorubicin, and taxanes were associated with variable incidences of cognitive impairment (from 3% to 62% across primary studies) in different cognitive domains
Hutchinson et al, 2012	24	Patients with BC and others	N/A	N/A	1274	N/A	CT	N/A	High dose or standard dose	Perceived vs objective assessment of cognition	N/A	None	Most primary studies involved patients with BC. Most studies did not find a significant relationship between objective and subjective measures of cognitive performance.
Ibrahim et al, 2021	13—cohort studies; case control studies	Patientswith BC; HC; patients with BC who didn’recieve chemotherapy	I to III	N/A	Age range: 18-69 years old; 100% females	Pre-menopausal; post-menopausal	CT (alone or with radiation therapy/endocrine therapy/target therapy)	N/A	Taxane based	Objective cognitive functioning	Attention and concentration, executive function, language, visual memory, verbal memory, processing of information, visuospatial and motor function.	Depression	Attention, concentration, depression, and executive function domains displayed CT-induced impairment. A noticeable, but not significant, impairment was observed for processing speed, visual memory, visuospatial and motor functions.
Jebahi et al, 2021	17—Cross-sectional; Longitudinal; RCT	BC patients; HC	N/A	HR+	1914 total subjects; mean age range: 43.66 to 68.30 years; 100% females	N/A	ET	Adjuvant	Tamoxifen (alone)	Objective cognitive functioning	Attention, memory, speed, executive functioning, verbal abilities, visual abilities, and language	None	Tamoxifen seemed to negatively affect cognitive functions such as immediate verbal memory and processing speed, regardless of the comparison group. Negative effects of tamoxifen on cognitive flexibility functions and verbal fluency were found to be only significant when comparing patients with BC to healthy controls or women with BC not undergoing any treatment.
Koumarianou et al, 2019	8—Cross-sectional, case control studies;	Patients with BC	I to III	N/A	1253 total subjects; age range:20 to > 70 years old; 100% females	Pre-menopause; post-menopause	CT	Adjuvant	N/A	Objective cognitive functioning; Subjective cognitive functioning	Attention, executive function, concentration, verbal and visual memory, working memory, processing speed, general intelligence, perceived cognitive abilities, impact on quality of life; work output	Stress	Stress seemed to mediate cognitive deficits in systemically treated patients, as shown by either self-completed questionnaires, neuropsychological testing or both. Such difficulties were reported to a lesser degree by Patients with BC not receiving CT. Older age was found to be associated with lower stress levels.
Lee et al, 2016	21	Patients with BC	N/A	HR+	2398 total subjects; mean age: 55.8 years; 100% females	Pre-menopause; post-menopause	ET	Adjuvant	N/A	Objective cognitive functioning	Learning/ memory; processing speed/information processing; executive function; language	none	ET was associated with impaired performance on neuropsychological testing in different domains (learning/memory, processing speed, executive functioning and language). No study explored the role of age.
Pullens et al, 2010	27—cross-sectional; longitudinal: RCT	Patientswith BC; HC; women with BC who didn’t recieve chemotherapy	N/A	N/A	Sample size range: 21-2433 subjects; mean age: N/A; 100% females	Pre-menopause; post-menopause	CT; ET	Adjuvant; neoadjuvant	N/A	Subjective cognitive functioning	N/A	Psychological distress; fatigue	21%-90% of the patients-reported SCD, mainly regarding memory, concentration, language, and self-reported retardation in mental processes or lower effectiveness. SCD and OCD were unrelated, but SCD was associated with psychological distress, fatigue, and health status.
Sousa et al, 2020	16	Patientswith BC; HC; women with BC who didn’t recieve chemotherapy	I to III	N/A	949 total subjects; mean age: 50.8 tears old; 100% females	Pre-menopause; post-menopause	CT	Adjuvant (*N* = 14); neoadjuvant (*N* = 2)	Standard-dose polychemotherapy regimens, with the majority of patients receiving a combination of doxorubicin, cyclophosphamide, and paclitaxel	Objective cognitive functioning; subjective cognitive functioning	Visual memory; working memory; verbal memory; episodic memory; processing speed; attention; executive functioning; distractibility; verbal fluency	Anxiety and depression; QoL; fatigue; worry; percieved stress; trauma personality; anger, confusion	CT was not associated with cognitive side effects. Long-term assessments showed improved verbal memory and processing speed, although patients often reported cognitive impairment. Brain abnormalities were detected during treatment, peaked after CT, and partially recovered over time. No significant differences in anxiety and depression were found, but there were clinically significant fatigue symptoms in the CT group, especially in patients reporting subjective cognitive difficulties.
Underwood et al, 2018	14—cross-sectional; longitudinal	Patientswith BC; women with BC who didn’t recieve endocrine therapy; HC	I-III	HR+	1822 total subjects; mean age range: 44 to 68 years; 100% females	Pre-menopause; post-menopause	ET	N/A	Tamoxifen; Aromatase Inhibitors (steroidal: ie, anastrozole; OR non-steroidal: ie, exemestane; letrozole) OR mixed)	Objective cognitive functioning	Verbal learning/memory, visual learning/memory, processing speed, frontal executive function, psychomotor efciency, and visuospatial function	Depression and anxiety	Patients undergoing ET performed worse than controls on verbal and visual learning/memory, executive function, and processing speed, but not on psychomotor efficiency or visuospatial function. Tamoxifen and aromatase inhibitors patients did not differ from one another, although tamoxifen patients performed better than non-steroidal aromatase inhibitors patients. Most studies reported no significant differences in anxiety or depression.
Whittaker et al, 2022	52—cross-sectional; longitudinal, cohort studies, RCT	Patients with BC	I to III	N/A	26.692 total subjects; mean age: 52 years old; 100% females	Pre-menopause; post-menopause	CT (alone or in combination with hormone therapy/radiotherapy)	N/A	Common combinations: FEC; CMF; doxorubicin + paclitaxel, doxorubicin + cyclophosphamide	Objective cognitive functioning; subjective cognitive functioning	Global cognitive functioning; Executive functions; Language, Motor functioning; Processing Speed, Verbal/Visual Learning and Memory; Visuo-Spatial Function; Working Memory	none	Mean prevalence rates for CICI across all time-points were 44% using self-report and 6% using short cognitive screening. Prevalence of CRCI was higher when based on patients’ self-reported experience rather than neuropsychological test results.
Yao et al, 2017	N = 41—cross-sectional; longitudinal	Patientswith BC; HC; Women with BC who didn’t recieve chemotherapy	N/A	N/A	5295 subjects; mean age: N/A; 100% females	N/A	CT	N/A	N/A	Objective cognitive functioning	Executive functions (inhibition, shifting and updating)	None	Inhibition appears relatively spared from the effects of CT, whereas impairments in shifting and updating are more commonly found following CT.

Abbreviations: BC, breast cancer; CT, chemotherapy; ET, endocrine therapy; HC, healthy controls; QoL, quality of life; RT, radiation therapy; FEC, (5-fuorouracil, epirubicin, and cyclophosphamide); CMF, (cyclophosphamide, methotrexate and 5-fuorouracil), doxorubicin + paclitaxel, doxorubicin + cyclophosphamide.

### Objective cognitive functioning

Eleven systematic reviews exclusively investigated objective cognitive functioning as assessed by standardized tests. Six reviews investigated the cognitive effects of chemotherapy.

One review^30^ showed that compared to baseline assessments or healthy control data, attention, concentration, and executive functions were significantly impaired in patients treated with taxane-based chemotherapy.

Similarly, Huehnchen et al^31^ found evidence that combinations of 5-fluorouracil, epirubicin, cyclophosphamide, doxorubicin, and taxanes were associated with cognitive impairment in multiple cognitive domains. Another review^32^ found the presence of cognitive impairment in Patients with BC treated with chemotherapy, especially concerning verbal memory, processing speed, and executive functioning.

In the same line, Di Iulio et al^33^ found that chemotherapy, alone and combined with hormonal therapy, influenced in particular fluency, memory, executive function, and motor speed.

Similarly, Cerulla-Torrente et al^34^ found cognitive impairment in attention, memory, executive functions, and processing speed. Cognitive impairment was frequent at the end of chemotherapy, and it seemed to gradually improve 1-2 years after treatment.

Finally, Yao et al^35^ focused on executive functioning (ie, inhibition, shifting, and updating), finding that inhibition appears spared from the effects of chemotherapy, whereas impairments in shifting and updating were more commonly found across studies.

Four systematic reviews focused on endocrine therapy. Lee et al^36^ found that endocrine therapy was associated with impaired performance in learning/memory, processing speed, executive functioning, and language.

Another review^37^ revealed that endocrine therapies were accompanied by altered cognitive abilities, including verbal memory, fluency, motor speed, attention, and working memory. Tamoxifen was related to decreased cognitive performances compared with aromatase inhibitors.

Similarly, Jebahi et al^38^ found that tamoxifen negatively affected cognitive performance on immediate verbal memory and processing speed.

In another review,^39^ patients undergoing endocrine therapy performed worse than control groups on verbal and visual learning/memory, executive function, and processing speed. In this case, tamoxifen patients performed better than non-steroidal aromatase inhibitor recipients.

Finally, Dijkshroon et al,^40^ compared the effects of chemotherapy to the effects of hormonal therapy on cognition. Patients undergoing chemotherapy seemed to have a higher chance of cognitive decline.

Summarizing, all systematic reviews found significant cognitive impairment following chemotherapy and endocrine therapy in patients with BC, particularly in those treated with taxanes and tamoxifen. Memory, attention/concentration, executive functioning, and processing speed were the most affected cognitive domains.

### Association between objective cognitive functioning and subjective cognitive functioning

Seven systematic reviews investigated the relationship between objective cognitive functioning and subjective cognitive concerns following chemotherapy.

Papanastasiou et al^41^ examined the impact of stress, age, and adjuvant chemotherapy on cognitive impairment in patients with BC. Results revealed a significant association between stress and cognitive dysfunction, as shown through self-report questionnaires and neuropsychological tests.

Differently, another review^42^ found that chemotherapy was not associated with short-term cognitive side effects. Long-term assessments showed improved verbal memory and processing speed. However, patients often reported subjective cognitive concerns.

Whittaker et al^43^ examined chemotherapy-induced cognitive impairment, as ascertained using self-report measures and neuropsychological tests. Results revealed that the prevalence of CRCI was higher when based on patients’ self-reported experience rather than neuropsychological test results.

Hutchinson et al^44^ found that most studies did not show a significant relationship between objective and subjective measures of cognitive performance.

Pullens et al^24^ compared chemotherapy and hormonal therapy, finding that subjective cognitive concerns were not related to objective neuropsychological test results.

Similarly, Bray et al ^45^ did not find an association between self-reported cognitive symptoms and neuropsychological results in most of the primary studies.

Summarizing, most reviews evidenced that subjective cognitive concerns were weakly or not associated with objective cognitive functioning as measured by neuropsychological test results.

### Other associated outcomes

Seven systematic reviews considered psycho-physical factors when examining cancer treatments’ effects on cognition. Most of them focused on chemotherapy’s effects, while one investigated endocrine therapy.

In one review,^24^ psychological distress and fatigue were examined in their relationship with subjective cognitive concerns. These were not related to objective test results but were associated with psychological distress, fatigue, and health status.

Sousa et al^42^ observed clinically significant fatigue symptoms in the chemotherapy group. Patients reporting subjective cognitive concerns were also more likely to experience depression, worry, and fatigue.

Similarly, in Underwood et al^39^ most studies that compared endocrine therapy patients and healthy controls reported no significant differences in measures of anxiety or depression.

Conversely, another review^30^ evidenced that patients with BC treated with chemotherapy displayed higher levels of depression, compared to their baseline assessment or healthy controls.

Concerning the quality of life, Dweck et al ^32^ found that the level of cognitive decline following chemotherapy did not consistently affect patients’ quality of life.

Concerning stress, generalized stress, job stress, and post-traumatic symptoms mediated cognitive deficits in systemically treated patients. In older patients, lower stress levels were associated with better cognitive function.^41^

Finally, another review^46^ identified a relationship between modifiable biological, behavioral, and psychosocial factors and subjective/objectively measured cognitive deficits.

Summarizing, most reviews evidenced a relationship between cognitive impairment and psychological distress, fatigue, poor sleep quality, and inflammatory and biological factors after chemotherapy and endocrine therapy.

### Quality assessment of included systematic reviews

Methodological quality was evaluated with the AMSTAR 2 (Table S2, Supplementary materials). Included systematic reviews were rated as having critically low (*n* = 8), low (*N* = 1), moderate (*n* = 6), and high (*n* = 3) methodological quality.

## Discussion

This umbrella review aims to provide a comprehensive and up-to-date overview of the evidence concerning the cognitive impact of various BC therapies. A secondary aim is to examine how objective cognitive deficits, and subjective cognitive concerns, alongside other psycho-physical factors, contribute to the development and persistence of CRCI. While research into the cognitive effects of cancer treatments is expanding, evidence remains scattered due to differences in study methodologies, limited data on new therapies, and long-term effects in life-long treated patients. Addressing these issues would help to uniformly address the unmet needs of patients with BC dealing with CRCI.

### Cognitive impairment following breast cancer therapies

Overall, results suggest that chemotherapy and endocrine therapy are often associated with CRCI in BC patients with BC.^39,47-50^ Although possible pathophysiological mechanisms are still under investigation, chemotherapy-induced cognitive impairment is believed to have a multifactorial etiology: different molecular mechanisms may result in blood-brain barrier disruption, systemic and chronic inflammation, accelerated cellular senescence, and neuronal stem cell abnormalities, all of which can potentially lead to cognitive impairment.^18^ Regarding endocrine therapy, it appears that verbal memory^51^ and executive functions^52^ are particularly sensitive to the effects of estradiol, likely due to the distribution of estrogen receptors in the brain. Endocrine therapy might therefore directly affect cognition by influencing oestradiol signaling but could also have indirect effects on cognition mediated by the presence of other side effects such as fatigue, sleep disturbance, depression, and anxiety.^25^ Overall, these findings suggest that cancer therapies can influence cognition potentially through a combination of direct and indirect effects on the nervous system.^18^

Across the included reviews, memory, attention/concentration, executive functioning, and processing speed were the most affected cognitive domains. This pattern was found for both chemotherapy and endocrine therapy recipients and is in line with the current literature These observations define a frontal-subcortical cognitive network,^53^ which is further corroborated by neuroimaging studies demonstrating brain alterations post-chemotherapy and endocrine therapy.^18,54,55^ Notably, the presence of similar impairment profiles in both chemotherapy and endocrine therapy-treated patients suggests a transversal etiology of CRCI. Nonetheless, chemotherapy may have endocrine effects in pre-menopausal patients and chemotherapy recipients often undergo endocrine therapy as well, making it challenging to isolate individual effects.

Our results also showed that CRCI was particularly enhanced in those treated with taxane-based chemotherapy and tamoxifen. As taxanes can cross the blood-brain barrier and accumulate in the brain they have been associated with molecular changes in the central nervous system.^56^ This has been linked to destabilization of the neuronal structure (ie, alteration in hippocampal functions, loss of spines, and dendritic arborization) and impaired neurotransmission, resulting in impaired cortex-based task performance.^57-59^ As regards tamoxifen, studies generally indicate that it is often associated with worsening cognitive functioning.^60^ However, despite the well‐known link between estrogen and brain tissue functioning, evidence is mixed, with some studies showing no effects on cognition.^61^ As tamoxifen is a selective estrogen receptor modulator, it can act both as an agonist and antagonist in various parts of the body, therefore giving rise to heterogeneous patterns of cognitive changes.^62^

Overall, certain limitations stem from the included systematic reviews. Primarily, the heterogeneity in study design and methodology across the reviewed studies poses substantial obstacles in interpreting the results. Additionally, a wide array of neuropsychological tests and questionnaires was used, possibly contributing to differences in study results. Although most studies shared an overlapping set of neuropsychological tests (ie, Trail Making Test, Controlled Oral Word Association Test, Rey–Osterrieth Complex Figure Test, Stroop Test, and California Verbal Learning Test) a total of 124 different neuropsychological tests were used in the primary studies (Table S3, Supplementary material). Besides, language was investigated only in a minority of studies and was often limited to verbal fluency. The heterogeneity in the selection of neuropsychological tests and definitions of cognitive impairment highlights the ongoing lack of consensus regarding which specific neuropsychological tests are most suitable for evaluating cognitive functioning in this clinical population. Furthermore, many reviews failed to consider critical information, (ie, cancer molecular type, pharmacological details, treatment setting, and menopausal status), which may impact the comprehensiveness of the conclusions drawn. Finally, the included reviews exclusively investigated the effect of chemotherapy and endocrine therapy on cognition, while no systematic review concerned immunotherapies cognitive effects. As CRCI also seems to exist with targeted therapies and immunotherapy,^63,64^ further research is needed due to potential neurotoxicity and the increasing prominence of these treatments in oncology.^65^

### Relationship between subjective cognitive concerns, objective cognitive deficits, and other mediating factors

In line with the existing literature,^26,44,66,67^ the present umbrella review found a weak association between subjective cognitive concerns and neuropsychological test results, with a higher prevalence of CRCI when considering patients’ subjective cognitive concerns. Several factors might account for this phenomenon, which are not mutually exclusive:

1. Variability in the choice of neuropsychological tests and definitions of cognitive impairment across studies could contribute to the mixed results, as different studies used multiple measures for similar constructs (eg, working memory performance was defined with the PASAT score in some studies, whereas in others with the TMT-B score).
2. Traditional neuropsychological tests may lack the sensitivity and specificity required to detect subtle cognitive changes in this clinical population. Additionally, given that CRCI symptoms can fluctuate, they might not always be discernible during objective neuropsychological assessments.^68^ In contrast, subjective cognitive concerns often encompass patients’ experiences over a more extended period,^69^ potentially leading to higher reported rates.
3. Subjective and objective measures of cognitive functions may evaluate different constructs, and perceived cognitive difficulties may reflect psychological distress rather than actual cognitive impairment.^45^

Consistently with prior evidence,^70-73^ results evidenced a positive relationship between CRCI and other psycho-physical factors in patients with BC undergoing chemotherapy and endocrine therapy. Interestingly, it has been pointed out that subjective cognitive concerns exhibit a stronger correlation with other psychological and physical symptoms rather than with the results of neuropsychological tests.^18,74^ While the precise nature of these relationships remains unclear, it is apparent that CRCI is a multifaceted phenomenon influenced by many factors that may collectively contribute to the onset and persistence of cognitive impairment.

These encompass demographic characteristics,^75^ increased levels of circulating cytokines,^76,77^ and inflammation,^78^ as well as the stage and characteristics of the disease, the type of treatment received. Other aspects include lifestyle factors like diet and exercise,^79–81^ and co-occurring psychological distress, fatigue, and sleep disorders.^24^ All these elements may independently contribute to CRCI but also exert mutual influence, either predisposing individuals to CRCI or perpetuating its effects. For instance, cancer diagnosis and treatments can lead to emotional distress, anxiety, and depression, which can adversely impact cognitive functions.^19^ These emotional states can influence neural signaling via neuroinflammatory processes and disruption of the hypothalamic-pituitary axis, potentially resulting in impaired neuroplasticity and cognitive functioning.^82^ Moreover, intrusive thoughts related to the traumatic experience can consume attentional resources, negatively affecting working and episodic memory.^83^ Concurrently, experiencing cognitive difficulties may exacerbate emotional distress, fatigue, and sleep disturbances, setting in motion a detrimental cycle where each symptom exacerbates the others.^18^

Therefore, it is important to consider all these factors and their complex interplay when investigating CRCI.^84^ As no study adopted a holistic approach that examined all these variables together, this limitation hinders our ability to disentangle the intricate relationships among these variables. Characterizing the complete array of symptoms could offer valuable insights into the mechanisms that perpetuate cognitive impairment following cancer treatments.

### Conclusion and future perspectives

The present review suggests that chemotherapy and endocrine therapy consistently appear to be associated with CRCI in patients with BC. Furthermore, subjective cognitive concerns appeared weakly or not associated with neuropsychological test results. Finally, CRCI was also consistently associated with psychological distress, fatigue, and sleep quality.

Overall, our results highlight the need for more systematic and harmonized studies on CRCI. Moreover, further research should explore the cognitive effects of immunotherapy and targeted therapies, which may have neurotoxic potential. Neuropsychological assessments conducted at multiple time-points should be incorporated to account for possible cognitive fluctuations. Furthermore, research should aim at providing a universally accepted battery of neuropsychological tests, with adequate and comprehensive normative data, that also considers other factors that might play a role in CRCI such as subjective cognitive concerns, information regarding mood, sleep, and other symptoms commonly experienced by patients with BC. Further studies are needed to elucidate the interplay among these factors and their precise roles in the manifestation of CRCI. This would provide valuable insights into the underlying mechanisms that sustain CRCI, ultimately enabling the design of tailored cognitive rehabilitation interventions^85^ that meet the individual needs of patients with BC.

## Supplementary Material

oyae090_suppl_Supplementary_Material

## Data Availability

The data underlying this article are available in the article and in its online supplementary material.
