# Supplementary material for: Cognitive impairment following breast cancer treatments: an umbrella review
Source: Oncologist. 2024 May 9;29(7):e848–63. doi: 10.1093/oncolo/oyae090 (PMC11224991; doi:10.1093/oncolo/oyae090)
Supplement: oyae090_suppl_Supplementary_Material [file oyae090_suppl_supplementary_material.docx]

**SUPPLEMENTARY MATERIALS**

**Table S1**. Search strings and number of results from the databases used in this review (i.e., Pubmed, Embase, and Scopus).

| **Database** | **Search string** | **Records** |
| --- | --- | --- |
| Pubmed | ((("breast cancer"[Title/Abstract]) OR ("breast neoplasm"[Title/Abstract]) OR ("mammary cancer"[Title/Abstract])) AND ((chemotherapy[Title/Abstract]) OR (radiotherapy[Title/Abstract]) OR ("endocrine therapy"[Title/Abstract]) OR ("hormonal therapy"[Title/Abstract]) OR ("immunotherapy"[Title/Abstract])) AND ((cognit*[Title/Abstract]) OR (neuropsycholog*[Title/Abstract])) AND (systematic review[Filter])) | 39 |
| Embase | ('breast cancer':ab,ti OR 'breast neoplasm':ab,ti OR 'mammary cancer':ab,ti) AND (chemotherapy:ab,ti OR radiotherapy:ab,ti OR 'endocrine therapy':ab,ti OR 'hormonal therapy':ab,ti OR immunotherapy:ab,ti) AND (cognit*:ab,ti OR neuropsychol*:ab,ti) AND 'systematic review':ab,ti | 58 |
| Scopus | TITLE-ABS-KEY ( "breast cancer" ) OR TITLE-ABS-KEY ( "breast neoplasm" ) OR TITLE-ABS-KEY ( "mammary cancer" ) AND TITLE-ABS-KEY ( chemotherapy ) OR TITLE-ABS-KEY ( radiotherapy ) OR TITLE-ABS-KEY ( "endocrine therapy" ) OR TITLE-ABS-KEY ( "hormonal therapy" ) OR TITLE-ABS-KEY ( immunotherapy ) AND TITLE-ABS-KEY ( cognit* ) OR TITLE-ABS-KEY ( neuropsychol* ) AND TITLE-ABS-KEY ( "systematic review" ) | 169 |

**Table S2.** Quality assessment.

Methodological quality was evaluated using the AMSTAR 2 tool (Shea et al 2017), which assesses the methodological quality of systematic reviews that include randomized and non-randomized studies of healthcare interventions. AMSTAR 2 includes the following critical domains: protocol registered before the start of review; adequacy of literature search; justification for excluded studies; risk of bias for included studies; appropriateness of meta-analytic methods; consideration of the risk of bias when interpreting results; and assessing the presence and likely impact of publication bias (Shea et al 2017). The tool provides guidance to rate the overall confidence in the results of a review (high, moderate, low, or critically low depending on the number of critical flaws and/or non-critical weaknesses). As the majority of included systematic reviews did not include a metanalysis, items 2, 11, and 15 were not considered critical.

| **Authors** | **Item 1** | **Item 2** | **Item 3** | **Item 4*** | **Item 5** | **Item 6** | **Item 7*** | **Item 8** | **Item 9*** | **Item 10** | **Item 11** | **Item 12** | **Item 13*** | **Item 14** | **Item 15** | **Item 16** | **AMSTAR 2 rating** |
| --- | --- | --- | --- | --- | --- | --- | --- | --- | --- | --- | --- | --- | --- | --- | --- | --- | --- |
| Bakoyiannis et al., 2016 | Yes | Partial yes | No | Partial yes | Yes | Yes | Yes | Yes | No | No | N/A | N/A | No | Yes | N/A | Yes | Critically low |
| Bray et al., 2018 | Yes | No | Yes | No | Yes | Yes | Yes | Yes | No | No | N/A | N/A | No | Yes | N/A | Yes | Critically low |
| Cerulla Torrente et al., 2020 | Yes | No | Yes | Yes | No | No | Yes | Partial yes | No | No | N/A | N/A | No | Yes | N/A | Yes | Critically low |
| Di Iulio et al., 2019 | Yes | No | No | Partial Yes | Yes | Yes | Yes | Partial yes | No | No | N/A | N/A | No | Yes | N/A | Yes | Critically low |
| Dijkshoorn et al., 2021 | Yes | Partial yes | Yes | Partial yes | Yes | Yes | Yes | Partial yes | Yes | No | N/A | N/A | Yes | Yes | N/A | Yes | High |
| Dwek et al., 2017 | Yes | No | No | Partial yes | No | No | Yes | Partial yes | Partial yes | No | N/A | N/A | Yes | Yes | N/A | Yes | Moderate |
| Henneghan, 2016 | No | No | No | Partial yes | No | No | No | Partial yes | Yes | No | N/A | N/A | Yes | Yes | N/A | Yes | Low |
| Huehnchen et al., 2020 | No | No | Yes | No | No | No | Yes | Partial yes | No | No | N/A | N/A | No | Yes | N/A | Yes | Critically low |
| Hutchinson et al., 2012 | Yes | No | Yes | Partial yes | No | No | Partial yes | Partial yes | No | No | N/A | N/A | No | Yes | N/A | Yes | Critically low |
| Ibrahim et al., 2021 | Yes | Yes | No | Partial Yes | Yes | Yes | Yes | Partial yes | Yes | No | Yes | Yes | Yes | Yes | Yes | Yes | Moderate |
| Jebahi et al., 2021 | Yes | Partial yes | No | Yes | Yes | Yes | Yes | Partial yes | No | No | N/A | N/A | No | Yes | N/A | Yes | Critically low |
| Papanastasiou et al., 2019 | Yes | No | Yes | Partial yes | Yes | Yes | Yes | Partial yes | Yes | No | N/A | N/A | Yes | Yes | N/A | Yes | Moderate |
| Lee et al., 2016 | Yes | No | No | Yes | No | No | Yes | Partial yes | Yes | No | N/A | N/A | Yes | Yes | N/A | Yes | Moderate |
| Pullens et al., 2010 | Yes | No | No | Partial yes | No | No | Partial yes | Partial yes | Yes | No | N/A | N/A | Yes | Yes | N/A | Yes | Moderate |
| Sousa et al., 2020 | Yes | Yes | Yes | Partial Yes | Yes | Yes | Yes | Partial yes | Partial yes | No | N/A | N/A | Yes | Yes | N/A | Yes | High |
| Underwood et al., 2018 | Yes | No | No | Yes | Yes | Yes | Yes | Yes | Partial yes | No | Yes | No | Yes | Yes | Yes | Yes | Moderate |
| Whittaker et al., 2022 | Yes | Yes | Yes | Partial yes | Yes | Yes | Yes | Partial yes | Partial yes | No | Yes | Yes | Yes | Yes | Yes | Yes | High |
| Yao et al., 2017 | Yes | No | No | Yes | Yes | Yes | Partial yes | Partial yes | No | No | N/A | N/A | No | Yes | N/A | Yes | Critically low |

***Legenda:*** N/A: not applicable; *: critical domain

**Items:** Item 1: Did the research questions and inclusion criteria for the review include the components of PICO? Item 2: Did the report of the review contain an explicit statement that the review methods were established prior to the conduct of the review and did the report justify any significant deviations from the protocol? Item 3: Did the review authors explain their selection of the study designs for inclusion in the review? Item 4: Did the review authors use a comprehensive literature search strategy? Item 5: Did the review authors perform study selection in duplicate? Item 6: Did the review authors perform data extraction in duplicate? Item 7: Did the review authors provide a list of excluded studies and justify the exclusions? Item 8: Did the review authors describe the included studies in adequate detail? Item 9: Did the review authors use a satisfactory technique for assessing the risk of bias (RoB) in individual studies that were included in the review? (RCT/NRSI) Item 10: Did the review authors report on the sources of funding for the studies included in the review? Item 11: If meta-analysis was performed did the review authors use appropriate methods for statistical combination of results? (RCT/NRSI) Item 12: If meta-analysis was performed, did the review authors assess the potential impact of RoB in individual studies on the results of the meta-analysis or other evidence synthesis? Item 13: Did the review authors account for RoB in individual studies when interpreting/ discussing the results of the review? Item 14: Did the review authors provide a satisfactory explanation for, and discussion of, any heterogeneity observed in the results of the review? Item 15: If they performed quantitative synthesis did the review authors carry out an adequate investigation of publication bias (small study bias) and discuss its likely impact on the results of the review? Item 16: Did the review authors report any potential sources of conflict of interest, including any funding they received for conducting the review?

**Quality rating:**

**High** - Zero or one non-critical weakness: The systematic review provides an accurate and comprehensive summary of the results of the available studies that address the question of interest.

**Moderate** - More than one non-critical weakness: The systematic review has more than one weakness, but no critical flaws. It may provide an accurate summary of the results of the available studies included in the review.

**Low**- One critical flaw with or without non-critical weaknesses: The review has a critical flaw and may not provide an accurate and comprehensive summary of the available studies that address the question of interest.

**Critically low** - More than one critical flaw with or without non-critical weaknesses: The review has more than one critical flaw and should not be relied on to provide an accurate and comprehensive summary of the available studies.

Reference:

Shea BJ, Reeves BC, Wells G, Thuku M, Hamel C, Moran J, Moher D, Tugwell P, Welch V, Kristjansson E, Henry DA. AMSTAR 2: a critical appraisal tool for systematic reviews that include randomised or non-randomised studies of healthcare interventions, or both. BMJ. 2017 Sep 21;358:j4008.

**Table S3.** Number of included reviews in which primary studies included the specific neuropsychological test or self-report questionnaire.

*Legenda:* WAIS: Wechsler Adult Intelligence Scale; WASI: Wechsler Abbreviated Scale Intelligence; WMS: Wechsler Memory Scale; WMS-R: Wechsler Memory Scale- revised

| Objective neuropsychological test | N. |
| --- | --- |
| Trail Making Test | 17 |
| Stroop Test | 14 |
| Central Nervous System Vital Signs | 13 |
| Controlled Oral Word Association Test | 12 |
| Repeatable Battery of the Assessment of the Neuropsychological Status | 12 |
| Rey–Osterrieth Complex Figure Test | 12 |
| California Verbal Learning Test | 11 |
| Digit-Symbol Coding (WAIS-III) | 11 |
| Rey Auditory Verbal Learning Test | 10 |
| Digit Span | 9 |
| Hopkins Verbal Learning Test | 9 |
| Wechsler Memory Scale (WMS–III) | 9 |
| Continuous Performance Test | 8 |
| Letter‐Number‐Sequencing, WAIS‐III | 8 |
| Logical Memory (WMS-R) | 8 |
| Paced Auditory Serial Addition Task | 7 |
| Delis-Kaplan Executive Function System | 6 |
| Finger Tapping | 6 |
| Grooved Pegboard | 6 |
| Visual Reproduction (WMS-R) | 6 |
| Boston Naming Test | 5 |
| Wisconsin Card Sorting Test | 5 |
| Auditory Consonant Trigrams | 4 |
| Block Design, WAIS‐III | 4 |
| Brief Visuospatial Memory Test Revised | 4 |
| Doors & People: verbal recall, verbal recognition, visual recall, visual recognition | 4 |
| Family Pictures (WMS-III) | 4 |
| Fepsy Finger Tapping | 4 |
| Fepsy Reaction Times | 4 |
| High Sensitivity Cognitive Screen | 4 |
| Kendrick Digit Copying Task | 4 |
| Rey Visual Learning Test | 4 |
| Rivermead Behavioral Memory Test | 4 |
| Symbol Search (WAIS-III) | 4 |
| Verbal fluency | 4 |
| Vocabulary (WASI) | 4 |
| Arithmetic (WAIS‐III) | 3 |
| Auditory verbal learning test | 3 |
| Benton Visual Retention Test | 3 |
| Concept Shifting test | 3 |
| Cognitive Stability Index (memory, processing speed and response speed) | 3 |
| Paragraph Recall subtest (WMS‐R) | 3 |
| Regensburger Wort-Flüssigkeits-Test | 3 |
| Test of Attentional Performance | 3 |
| Visual Association Test | 3 |
| Wechsler Adult Intelligence Scale (WAIS) | 3 |
| Behavioural Assessment of the Dysexecutive Syndrome | 2 |
| Booklet Category Test | 2 |
| California Computerized Assessment Package | 2 |
| Category Fluency | 2 |
| Concept shifting test | 2 |
| Conner ’s Continuous Performance Test | 2 |
| Cognitive Symptoms Checklist-Work (59 items) | 2 |
| D2 Test | 2 |
| Dual Attention to Response Task | 2 |
| Face Recognition Test (WMS‐III) | 2 |
| Faces (WMS-R) | 2 |
| Flanker Task | 2 |
| Game of Dice Task | 2 |
| Headminder computerized test | 2 |
| Iowa Gambling Task | 2 |
| Letter Digit Coding test | 2 |
| Letter cancellation task | 2 |
| Letter Fluency | 2 |
| list learning and recall | 2 |
| n‐back test | 2 |
| Object Location Memory | 2 |
| Shifting attention | 2 |
| Spatial Span Backward (WMS‐III) | 2 |
| Verbal Paired Associates I (WMS-R) | 2 |
| Visual Verbal Learning Test | 2 |
| Digit-Span (WAIS) | 2 |
| Nine hole peg test | 1 |
| Achievement Measure System | 1 |
| Animal Decoding | 1 |
| Attentive matrices test | 1 |
| Beck Depression Inventory | 1 |
| Benton Judgment of Line Orientation | 1 |
| Brown Location Test | 1 |
| Buschke Selective Reminding Test | 1 |
| Cambridge Neuropsychological Test Automated Battery | 1 |
| Clock drawing test | 1 |
| Comprehensive Trail Making Test | 1 |
| Continuous Paired Associate Learning Task | 1 |
| Design Organization Test | 1 |
| Digit Vigilance Test | 1 |
| Embedded Figures Test | 1 |
| Figural Memory (WMS-R) | 1 |
| Four Word Short Memory Test | 1 |
| Fepsy visual reaction test | 1 |
| Figural visual scanning test | 1 |
| Groton Maze Learning Test | 1 |
| Hamburger Wechsler Intelligence Test for Adults–Revised (HAWIE-R) | 1 |
| Hopkins Symptom Checklist | 1 |
| Intra-Extra Dimensional Set Shift task | 1 |
| Incidental Learning | 1 |
| International Shopping List Task | 1 |
| Lern- und Gedächtnistest (LGT-3) | 1 |
| Logical Memory Multiple Choice | 1 |
| Memory Cabinet | 1 |
| Memory for daily tasks | 1 |
| Mental Rotation Test | 1 |
| MMQ | 1 |
| Mini-Mental State Examination | 1 |
| Montreal Cognitive Assessment | 1 |
| One card Learning Task | 1 |
| Purdue Pegboard | 1 |
| Simbol digit modalities test | 1 |
| Story Memory | 1 |
| subtests from WAISS-III (working memory, verbal paired associate memory, narrative recall) | 1 |
| Symbol Scanning | 1 |
| Test of Everyday Attention | 1 |
| Visual Reproduction of the Wechsler Memory Scales-III (WMS-III) | 1 |
| Thumb-finger Sequencing | 1 |
| Tower of London | 1 |
| Visual attention | 1 |
| Visual Memory (WMS-R) | 1 |
| Visual Learning test | 1 |
| Verbal Learning and Memory Test | 1 |
| Visual Working Memory Test | 1 |

| Subjective cognitive concerns questionnaire | N. |
| --- | --- |
| European Organization for Research and Treatment of Cancer quality of life core questionnaire (EORTC-QLQ-C30) | 5 |
| Cognitive Failure Questionnaire (CFQ) | 4 |
| Patient’s Assessment of Own Functioning (PAOFI) | 4 |
| Structured/semi-structured interview | 4 |
| Attentional Function Index (AFI) | 3 |
| Functional Assessment of Cancer Therapy-Cognitive subscale (FACT-Cog) | 3 |
| Questionnaire for Self-perceived Deficits in Attention (FEDA) | 3 |
| Behavior Rating Inventory of Executive Function (BRIEF) | 2 |
| Broadbent cognitive failures questionnaire (BCFQ) | 2 |
| International Study Group of Postoperative Cognitive Dysfunction (ISPOCD) - questions about memory, concentration/ attention, mental burden and vitality | 2 |
| Multiple Ability Self-Report Questionnaire (MASQ) | 2 |
| Squire Memory Self-Rating Questionnaire (SSRQ) | 2 |
| Cognitive Functioning Scale | 2 |
| Cognitive stability index | 1 |
| European Organization for Research and Treatment of Cancer quality of life Breast Cancer Specific (EORTC QLQ-BR23) | 1 |
| Fatigue symptoms checklist | 1 |
| Multidimensional Fatigue Symptom Inventory -cognitive subscale (MFSI - cognitive) | 1 |
| Medical Outcomes Study Cognitive Scale (MOS-Cog) | 1 |
| Multidimensional Fatigue Inventory (MFI-20) | 1 |
| Quality of Life in Adult Cancer Survivors (QLACS) | 1 |
| Cognitive Symptoms Checklist-modified | 1 |
